# Influence of the Pyrolysis Temperature and TiO_2_-Incorporation on the Properties of SiOC/SiC Composites for Efficient Wastewater Treatment Applications

**DOI:** 10.3390/membranes12020175

**Published:** 2022-02-02

**Authors:** Natália C. Fontão, Lucas N. Ferrari, Joice C. Sapatieri, Kurosch Rezwan, Michaela Wilhelm

**Affiliations:** 1 Advanced Ceramics, University of Bremen, 28359 Bremen, Germany; fontao@uni-bremen.de (N.C.F.); ferrarii.lucas@gmail.com (L.N.F.); joicesapatieri@gmail.com (J.C.S.); krezwan@uni-bremen.de (K.R.); 2 Department of Mechanical Engineering, Federal University of Santa Catarina, Florianopolis 88040-900, Brazil; 3 Department of Chemical Engineering and Food Engineering, Federal University of Santa Catarina, Florianopolis 88040-900, Brazil; 4 MAPEX—Centre for Materials and Processes, University of Bremen, 28359 Bremen, Germany

**Keywords:** polymer-derived ceramics, methylene blue adsorption, microfiltration membranes, O/W emulsion separation

## Abstract

This study focuses on the development of porous ceramer and SiOC composites which are suitable for microfiltration applications, using a mixture of polysiloxanes as the preceramic precursor. The properties of the membranes—such as their pore size, hydrophilicity, specific surface area, and mechanical resistance—were tailored in a one-step process, according to the choice of pyrolysis temperatures (600–1000 °C) and the incorporation of micro- (SiC) and nanofillers (TiO_2_). Lower pyrolysis temperatures (<700 °C) allowed the incorporation of TiO_2_ in its photocatalytically active anatase phase, enabling the study of its photocatalytic decomposition. The produced materials showed low photocatalytic activity; however, a high adsorption capacity for methylene blue was observed, which could be suitable for dye-removal applications. The membrane performance was evaluated in terms of its maximum flexural strength, water permeation, and separation of an oil-in-water emulsion. The mechanical resistance increased with an increase of the pyrolysis temperature, as the preceramic precursor underwent the ceramization process. Water fluxes varying from 2.5 to 370 L/m^2^·h (2 bar) were obtained according to the membrane pore sizes and surface characteristics. Oil-rejection ratios of 81–98% were obtained at an initial oil concentration of 1000 mg/L, indicating a potential application of the produced PDC membranes in the treatment of oily wastewater.

## 1. Introduction

The rapid growth of the petrochemical, pharmaceutical, food and fertilizer industries has inevitably led to the intensive production of oily wastewater, which is one of the main sources of groundwater and surface water pollution [1]. This type of wastewater often contains micrometer-sized oil droplets dispersed in water, which form a stable oil-in-water emulsion even without a stabilizer, making the oil/water separation difficult using conventional processes (gravity separation, centrifugation, flocculation and coagulation) [2]. With growing environmental awareness worldwide, regulations, and the ever-increasing demand for clean water, the development of innovative and cost-effective technologies for water treatment has become a global concern [3,4]. In this scenario, membrane technology has emerged as a promising separation process for oily wastewater, as it offers a high separation efficiency and lower energy consumption, and is easy to scale-up [5,6].

Ceramic membranes have attracted more and more attention due to their outstanding properties, such as their high mechanical strength, their superior chemical/thermal resistance, the easy cleaning of membrane, and, consequently, their extended operating life [7]. However, their high manufacturing costs—especially that of the energy-intensive powder-sintering process—have limited their wider application [8,9]. As a suitable alternative to the conventional powder process, ceramic structures with compositions such as Si-O-C, Si-C and Si-(B)-N-C can be synthesized through the cross-linking and pyrolysis of a suitable polymeric precursor at lower processing temperatures than the ones required for the sintering process [10]. 

The general molecular structure of silicon-based polymers (-[Si(R1R2)-X]-) consists of an element/group X bonded to silicon (Si) atoms along the polymer backbone, which defines the class of Si-based materials, and the substituents R1 and R2 attached to silicon, which define the properties of the polymer, such as its chemical and thermal resistance, solubility, and rheology [11,12]. The main classes of Si-based materials are polysilanes (X = Si), polysiloxanes (X = O), polycarbosilanes (X = CH_2_), polysilazanes (X = NH) and polycarbodiimides (X = [N = C = N]) [13]. The bottom-up approach of the PDC route, using molecular compounds as a starting material, allows the control of the chemical composition and structure of the resulting ceramic, which is usually not possible with other techniques [10,14]. Additionally, polymer-derived ceramics (PDC) can be produced using polymer-forming techniques (fiber spinning, foaming, warm pressing, extrusion, injection molding or tape casting), allowing a near-net shape manufacturing of complex shapes, which can be later converted into the desired ceramic parts in an inert atmosphere [15,16,17]. 

At intermediate pyrolysis temperatures (500–700 °C), the decomposition of the organic functional groups in the preceramic polymer (and the resulting gas release) leads to an increase in porosity [18]. The resulting structure is a hybrid ceramic material with a high specific surface area [19]. However, a well-known drawback of PDC technology is the poor control of the shrinkage and structural integrity of the polymer-to-ceramic conversion products [20,21]. Thus, the developed transient porosity tends to disappear as the pyrolysis temperature increases. 

Pioneering studies [17,22,23,24] have shown that the incorporation of solid filler particles into the preceramic matrix can reduce the gas formation and the associated volume shrinkage during pyrolysis, thus reducing the formation of cracks and preventing the collapse of micro- and mesopores, resulting in a porous ceramic phase after the polymer-to-ceramic conversion. Aside from this, the addition of filler particles could provide additional functional properties such as hydrophilicity [25], high hardness/wear resistance [26], magnetic and electrical functionalities [27,28,29], and catalytic performance [30,31].

Silicon oxycarbides (SiOC) are among the best-researched PDC materials, as they have suitable mechanical properties and physical/chemical stabilities in high-temperature, oxidative and corrosive environments [32]. The SiOC structure consists of a network of corner-shared silicon-centered tetrahedra (Si–C and Si–O bonds) composed of a mixture of SiO_4_, SiO_3_C, SiO_2_C_2_, SiOC_3_ and SiC_4_, which remain predominantly amorphous at temperatures up to 1250 °C [33,34]. The versatility of the SiOC chemistry allows the properties to be modified for applications in various fields [35], such as gas separation [36,37], catalytic subtracts [12,38], and energy storage [39,40].

Despite the aforementioned advantageous properties, only a few studies have begun to evaluate the potential of PDC materials for microfiltration applications: Dong et al. [41] used a combination of polyhydromethylsiloxane and tetramethyl-tetravinyl-cycletetrasiloxane as a precursor to produce a ceramic membrane (pyrolyzed at 1200 °C) for the separation of an oil-in-water emulsion, while Zhang et al. [42] combined tetramethylcyclotetrasiloxane and tetramethyltetravinylcyclotetrasiloxane to develop a membrane (pyrolyzed at 1000 °C) for the filtration of rhodamine B. Those studies, however, did not investigate the potential of ceramer membranes for microfiltration applications using lower pyrolysis, or the possibility of tailoring the membrane properties—such as the hydrophilicity, specific surface area and mechanical resistance—in a one step-process, according to the choice of pyrolysis temperatures and different filler materials. Thus, this study focuses on the development of porous ceramer and SiOC composites tailored for microfiltration applications by investigating the effects of the pyrolysis temperature and the incorporation of micro- (SiC) and nanofillers (TiO_2_) on the PDC membrane morphology, pore size, porosity, surface characteristics, mechanical strength, and performance in terms of water permeation and the filtration of an oil-in-water emulsion. Additionally, the lower-temperature pyrolysis ranges (600–700 °C) could allow the incorporation of TiO_2_, in its active phase [31], such that the photocatalytic potential of the material could also be investigated.

## 2. Materials and Methods

### 2.1. Materials

Commercial methyl polysiloxane (Silres^®^ MK, Wacker Chemie AG, Burghausen, Germany) and phenylmethyl polysiloxane (Silres^®^ H44, Wacker Chemie) were used as the binders and preceramic polymer. Xylene (98.5%, Sigma–Aldrich, Hamburg, Germany) was the solvent for the polysiloxanes and the liquid medium, which was used to further disperse the components, and imidazole (99%, Imidazole) was applied as a cross-linking catalyst. Commercially available silicon carbide (SiC F800, d = 6.5 μm, Fluka) and titanium dioxide (TiO_2_, d = 21 nm, P25 Degussa) particles were incorporated as fillers. Azodicarbonamide (ADA, 97%, Sigma–Aldrich) was used as the pore former. Methylene blue (MB, Fluka) was used as a tracer dye. MCT oil was used to prepare an oil-in-water emulsion, and Polysorbate 80 (Tween 80, Sigma–Aldrich) was applied as a surfactant.

### 2.2. Membrane Processing Route 

**Figure 1:**
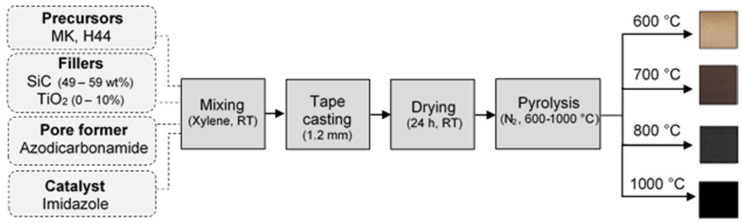
Flowchart of the membranes’ processing route.

The tape-casting technique was applied to produce porous hybrid ceramic membranes using a mixture of the methyl polysiloxanes (MK) and the phenylmethyl polysiloxane (H44) as preceramic precursors (ratio 1:1). The slurry preparation (Figure 1, Table S1) starts with the dissolving of the polysiloxanes in xylene, followed by the addition of azodicarbonamide to the solution under constant stirring (1 h). TiO_2_ particles were slowly added to the slurry in order to minimize agglomeration and ensure the homogeneity of the mixture (1 h). Then, the SiC particles were incorporated into the slurry and stirred for 1 h to produce a homogeneous mixture. The slurry was cross-linked using imidazole at room temperature (1 h) in order to avoid any later melting during heat treatment, cast over a polyethylene terephthalate carrier film (HOSTAPHAN^®^, Mitsubishi Polyester Film GmbH) using a doctor blade (gap = 1.2 mm), and dried at room temperature in a fume hood for 24 h. Afterwards, the dried green tapes were pyrolyzed at 600, 700, 800 and 1000 °C in order to evaluate the effect of the pyrolysis temperature (T_p_) on the membranes’ properties. In particular, lower pyrolysis temperatures could ensure that anatase is obtained as the phase of the TiO_2_ particles, which could allow some degree of photocatalytic activity of the membranes produced. The pyrolysis was performed in a nitrogen atmosphere (99.999% N_2_ purity), with a heating rate of 120 °C/h up to 100 °C below the final temperature (T_p_ –100 °C) and 30 °C /h until the final temperature (T_p_), with a dwelling time of 4 h. A cooling rate of 120 °C/h was applied at the end of the pyrolysis step. The samples were designated Tx_Siy-z, where x and y correspond to the respective percentage of the dry weight of TiO_2_ (0, 5 and 10%) and SiC (49, 54 and 59%) particles, and the pyrolysis temperature is given by z.

### 2.3. Characterizations 

The samples’ weight loss during pyrolytic conversion was investigated by thermogravimetric analysis (TGA, STA 503 Bähr), using a heating rate of 2 °C/min under a nitrogen flow (2 L/min). X-ray diffraction analysis (powder XRD, Seifert 3003) was conducted in order to identify the crystal phases of the pyrolyzed samples. The membrane macrostructure was analyzed by scanning electron microscopy (SEM, Zeiss EVO 10). Prior to the measurement, the samples were sputtered with gold (K550, Emitech, Judges Scientific, London, UK). The pore size distribution and the open porosity of the tapes were determined by mercury-intrusion porosimetry (Pascal 140/440 POROTEC). Nitrogen adsorption/desorption isotherm analysis performed at 77 K (Belsorp-Mini, Bel Japan, Osaka, Japan) determined the specific BET surface area (SSA). For these measurements, the pyrolyzed tapes were ground and sieved (x < 300 µm), and the powder was pretreated at 120 °C for 3 h in a vacuum. The surface characteristics of the membranes with regard to hydrophilicity/hydrophobicity were accessed by water and n-heptane vapor adsorption. For this analysis, the samples were ground, sieved (x < 300 µm), and dried at 70 °C (24 h). Then, each dried sample was placed in a vessel in an Erlenmeyer flask filled with water or heptane. The liquids were in equilibrium with their vapor phase at 20 °C. After 24 h, the samples were removed from the flasks and weighed, and the vapor uptake was determined. Adsorption/photocatalytic tests were performed using Methylene Blue as a tracer dye and a UV source (RSW-P03-365-0 3W UV LED, Roschwege GmbH, Greifenstein, Germany). The samples were ground, sieved (x < 300 µm), and brought into contact with an MB solution (C_0_ = 50 mg/L) under magnetic stirring. For comparison, these experiments were performed in the dark, or in the presence of the UV source. After certain time intervals, aliquots of 1 mL were taken from the stirred MB solutions, and centrifuged (12,000 rpm, 10 min) to remove the solid particles; the supernatants were then analyzed using a UV-Vis spectrophotometer (λmax = 665 nm). The maximum flexural strengths (σ_max_) of the tapes were obtained by three-point bending tests (Roell Z005, Zwick, Ulm, Germany). These measurements were performed using a 5 kN load cell (piezoelectric force sensor) at a fixed crosshead speed and a pre-load of 0.1 mm.min^−1^ and 0.25 N, respectively. Fifteen samples for each composition and temperature were cut into a rectangular format (16 mm length, 2 mm width, and 0.7–1.0 mm thickness) and placed in the center of a sample holder held by two cylindrical holders (d = 1.5 mm) 10 mm apart. The water permeation and oil-in-water (O/W) emulsion filtration performance of the membranes were assessed using a homemade setup in a dead-end configuration. Tapes of different compositions (T0 and T10) pyrolyzed at different temperatures were cut into a circular shape (10 mm diameter) and tested in duplicate at different pressures (1, 1.5 and 2 bar) for water permeation, and at 2 bar for the filtration experiments. The permeation flux was calculated according to Equation (1):

(1) $$J=\frac{1}{A}\frac{dm}{dt}$$

where *J* is the permeation flux (kg/m^2^·h), *A* is the transverse area of the membrane a (m^2^), *dm* represents the mass variation in kg, and *dt* is the time variation (h). DI water was used for the water permeation experiments, while filtration was carried out with an O/W emulsion using 1000 mg/L MCT oil. The size of the oil droplets was analyzed using a Laser Diffraction Particle Size Analyzer (Horiba LA-960, Hannover, Germany). Microfiltration was performed to a volume reduction factor of 2 (the ratio of initial feed volume to the final retentate volume). The oil removal was quantified by total organic carbon analysis (LCK386 kit, HACH, Düsseldorf, Germany), and the average oil rejection coefficients (*R*%) were calculated according to Equation (2) [32]:

(2) $$R\%=\left[1-\frac{C_{p}}{\left(\frac{C_{f0}-C_{rf}}{2}\right)}\right]\cdot 100\%$$

where *C_p_* is the raffinate concentration, *C_f_*_0_ is the initial feed concentration, and *C_rf_* corresponds to the final retentate in the batch system.

## 3. Results and Discussions

In this study, porous ceramer and ceramic membranes were produced via tape casting, using MK and H44 as preceramic precursors, azodicarbonamide as a pore formation agent, and imidazole as a cross-linking agent. The combination of methyl (MK) and phenylmethyl (H44) polysiloxanes in a ratio of 1:1 was a composition optimization previously established in our working group [43], which conferred sufficient mechanical stability to the tape-cast material. Additionally, the mixture of these polysiloxanes shows other advantages: because the decomposition of phenyl groups starts at lower temperatures (420–500 °C) than that of methyl groups (T > 600 °C), the use of H44 results in higher BET surface areas and lower hydrophobicity than MK when pyrolyzed at lower temperatures (500–600 °C) [18,44]. On the other hand, MK offers a higher ceramic yield (84 wt%) than H44 (72 wt%) under N_2_ at 1000 °C [45]. Complementary amounts of SiC (6.5 μm) and TiO_2_ (21 nm) were used as fillers in order to prevent the characteristic shrinkage of polysiloxanes-based material during the pyrolysis process, to increase mechanical stability, and to evaluate the effect of these micro- and nanofillers on the morphology, pore size, and surface characteristics of PDC membranes. The obtained slurries were homogenous, and no difficulties were observed during the tape-cast procedure. However, the formation of a TiO_2_ agglomerate cannot be excluded under these conditions. The produced tapes were pyrolyzed at 600, 700, 800 and 1000 °C, in an inert atmosphere (N_2_), in order to evaluate the effect of the temperature on the membrane properties. A gradual change in coloration was observed with the increase of the pyrolysis temperature. All of the pyrolyzed tapes presented sufficient handling stability. The membranes were evaluated in terms of their macro- and microstructure, surface characteristics, flexural strength, water permeation, and filtration of an oil-in-water emulsion.

Additionally, TiO_2_ is an efficient photocatalytic agent (under UV) which is usually used for the oxidative degradation of organic components in water treatment [31,33]. Apart from the aforementioned characteristics, the incorporation of TiO_2_ could impart a self-cleaning property to the PDC membranes (under an appropriate light source), which could reduce membrane fouling, one of the main obstacles in the application of membranes. This potential has mostly been explored in polymer membranes [31]. However, because the photocatalytic performance of TiO_2_ is phase-dependent—it shows a higher efficiency in the anatase phase (formation in the temperature range 400–700 °C) and a decreasing potential at the transition from the anatase to the rutile phase (complete conversion at around 1000 °C) [33]—this kind of application is not possible in a conventional ceramic membrane matrix, when TiO_2_ is applied as a starting powder, due to the high sintering temperatures applied in their fabrication (900–1700 °C) [34]. On the other hand, a pyrolysis temperature range of 600–700 °C could allow the incorporation of TiO_2_ in the anatase phase into the relatively thin membrane produced via the PDC processing route, and the photocatalytic potential of the material could thereby be investigated. 

### 3.1. Membrane Composition

According to the literature, the pyrolylitc decompositon of polysiloxanes at 1000 °C under nitrogen leads to the formation of SiOC [46,47,48]. A more detailed analysis of the structural changes during the pyrolytic decomposition of the methyl and methylphenyl polysiloxanes in N_2_ at 1000 °C can be found in the work of Cerny et al. [46]. Overall, the conversion of polysiloxanes to SiOC begins with the cross-linking of a preceramic precursor (100–400 °C), creating a siloxane polymer network. At pyrolysis temperatures above 400 °C, a series of radical reactions and rearrangements results in the cleavage of Si-C, C-C, Si-H, and C-H bonds. The authors [46] stated that the Si-O skeleton of the polymer is very stable, and should resist pyrolysis temperatures well above 1000 °C. Although redistribution reactions between Si-C and Si-O bonds are likely to occur during the pyrolysis at 1000 °C under N_2_, the number of Si-O bonds remains approximately constant. Additionally, these exchange reactions—in which Si-C bonds are exchanged for Si-O bonds and vice versa—are responsible for the formation of SiO_2_ and SiC nanodomains. An extensive study of the nanodomains in polymer-derived SiOC materials is described in the literature [49].

In this study, the decomposition behavior of the produced tapes was analyzed by thermal gravimetric analysis. Figure S1 shows the pyrolytic decomposition behavior of the different membrane compositions. The initial weight loss observed at temperatures between 100 and 400 °C is related to the degradation of the azodicarbonamide (200–300 °C), the cross-linking process of the preceramic polymers, and the consequent release/evaporation of the cross-linked products (water and alcohols), oligomers and solvents. MK has about 4% of the cross-linking active groups (-OH and -OR), while H44 has about 7%, and those groups are converted to H_2_O and alcohol (HOR) [18]. At temperatures above 400 °C, the weight loss results from the decomposition of the organic groups phenyl and methyl of preceramic polymers H44 and MK. A hybrid ceramic material (ceramer) is obtained using a pyrolysis temperature in the range of 400–800 °C, while SiOC structures are formed above 800 °C (ceramization process) [16].

**Figure 2:**
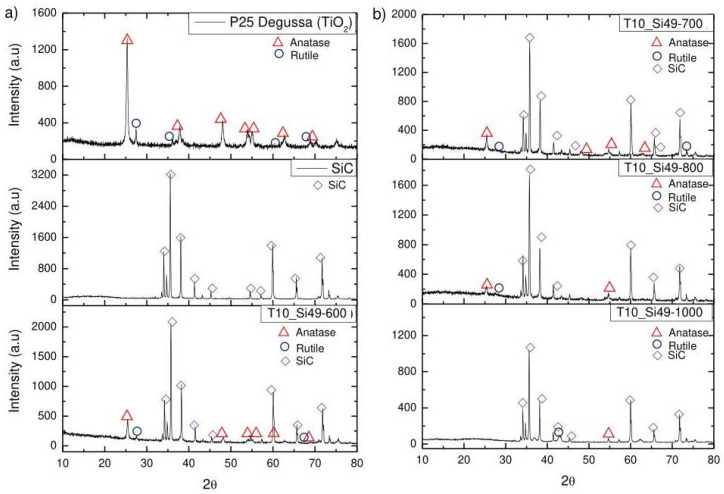
XRD analysis for the samples: (**a**) P25 Degussa (TiO_2_ powder), SiC powder and sample T10_Si49-600; (**b**) sample T10_Si49 pyrolyzed at different temperatures (700–1000 °C).

X-ray diffraction was used to determine the crystal structure of the produced membranes. For comparison, the pure TiO_2_ and SiC filler particles were also analyzed. Figure 2 shows the respective XRD spectra of TiO_2_ (P25 Degussa), SiC, and sample T10 pyrolyzed at different temperatures (600–1000 °C).

Silicon–oxycarbide (SiOC) material exhibits a remarkable resistance to crystallization, and its structures remain predominantly amorphous up to temperatures of 1250 °C [36]. Therefore, the peaks observed in the XRD spectra can be associated with the presence of TiO_2_ and SiC filler particles. The characteristic peak for anatase at 2θ = 25 and other anatase-related peaks in P25 Degussa were identified in the produced material pyrolyzed at different temperatures (600–800 °C). This confirms that TiO_2_ in the anatase phase was successfully incorporated into the membrane matrix. The increase of the pyrolysis temperature, however, promoted a reduction of the anatase peaks in the produced tapes, although some anatase peaks are still maintained in the samples pyrolyzed at 800 °C. Bhattacharjee et al. [37] observed a similar outcome when incorporating P25 Degussa TiO_2_ nanoparticles into an H44 foam. The authors suggested that the anatase nanoparticles were coupled to the Si-O backbone of the H44, forming titanosiloxane (Si-O-Ti-) bonds (further confirmed by FTIR spectroscopic investigation) that remain stable at higher temperatures (800 °C) than the usual range for the transformation of anatase to rutile (400–700 °C). However, Figure 2 shows that the remaining anatase peaks at 800 °C disappear with a further increase in pyrolysis up to 1000 °C. It is therefore to be expected that the photocatalytic potential of the samples produced will gradually decrease when the material is pyrolyzed at higher temperatures.

### 3.2. Membrane Macrostructure

**Figure 3:**
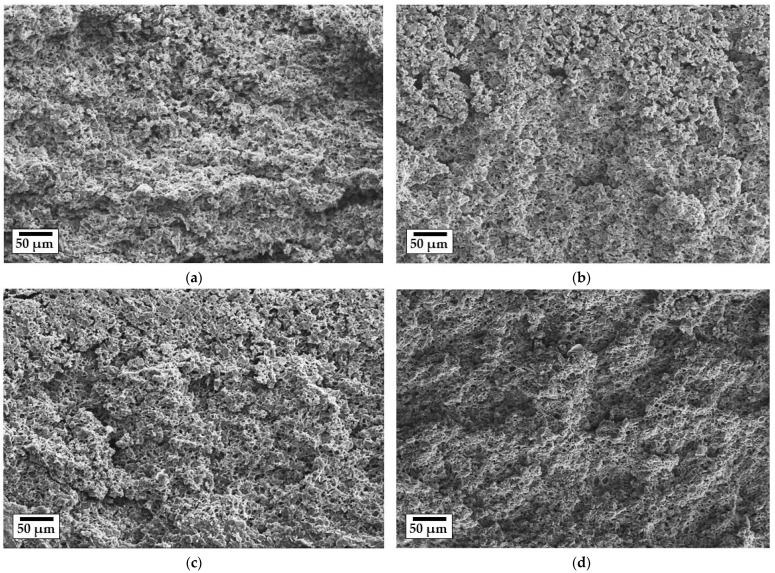
SEM images of the cross-section area of the membranes: (**a**) T0-Si59_600, (**b**) T10-Si49_600, (**c**) T10-Si49_800, and (**d**) T10-Si49_1000.

The macrostructures of the pyrolyzed tapes were analyzed by scanning electron microscopy and mercury intrusion porosimetry. All of the samples presented similar sponge-like structures (Figure S2) with small spheroidal pores homogeneously distributed in the matrix. Figure 3 shows a selection of membranes of different compositions pyrolyzed at different temperatures. The pore morphology was mainly determined by the degradation of the pore-forming agent azodicarbonamide during pyrolysis, as well as the space between the particles of different sizes, resulting in a network of interconnected pores in the membrane structure. 

The effect of the incorporation of ADA on polysiloxane-based tapes has been previously described by Nishihora et al. [50]. According to this investigation, the ADA—a typical blowing agent in a simultaneously cross-linking PDC [38]—is trapped by the cross-linked one, preventing the usual foaming process. The decomposition of ADA during the pyrolysis (300 °C) lead to a homogeneous porous structure constituted by small and irregularly shaped pores, as was also observed on the samples presented in Figure 3. A first comparison of the SEM images indicates that increasing the pyrolysis temperature or partially replacing the SiC with TiO_2_ particles does not seem to visibly affect the structure of the PDC matrix. Therefore, mercury intrusion analysis was performed to further investigate the membrane macrostructure. 

**Figure 4:**
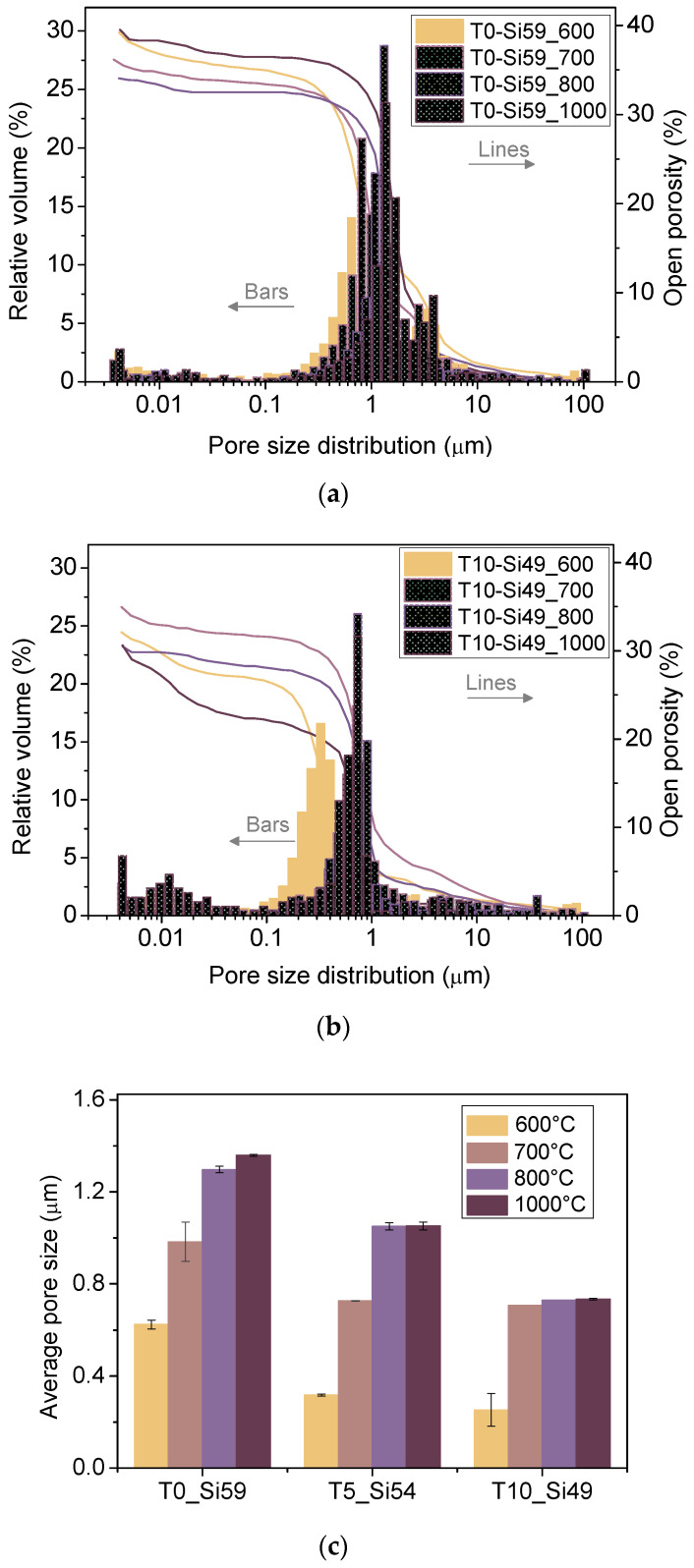
Pore size distribution, open porosity and average pore size for the tapes: (**a**) T0-Si59 and (**b**) T10_Si49 pyrolyzed at 600–1000 °C. (**c**) The average pore size for all of the samples pyrolyzed at 600–1000 °C.

Figure 4 presents the pore size distribution data for samples T0_Si59 and T10_Si49 pyrolyzed at different temperatures, and the average pore sizes for all of the samples obtained by mercury intrusion porosimetry. The sample T5_Si54 presented a pore-size distribution at intermediate values between T0_Si59 and T10_Si49; thus, they are not displayed. 

A narrow pore size distribution with an average pore size range of 0.3–1.4 μm and porosities of 30–40% was found for all of the samples. A general increase in pore size was observed with the increase in the pyrolysis temperature. In particular, a gradual decrease of the relative volume of the mesopores was observed as the pyrolysis temperature increased from 600 °C to 1000 °C. The collapse of micro/mesopores into larger pores at higher pyrolysis temperatures has already been reported in the literature [23]. On the other hand, increasing the amount of TiO_2_ resulted in smaller average pore sizes, which can be attributed to the size of the TiO_2_ nanoparticles (21 nm), which can aggregate and thereby fill empty voids in the material structure. Therefore, the average pore size of the microfiltration membranes can be tailored by adjusting the pyrolysis temperature and the filler content. Apart from this, thermal treatment and the incorporation of nanofillers can also affect other membrane properties, which was further investigated.

### 3.3. Microporosity and Surface Characteristics

**Figure 5:**
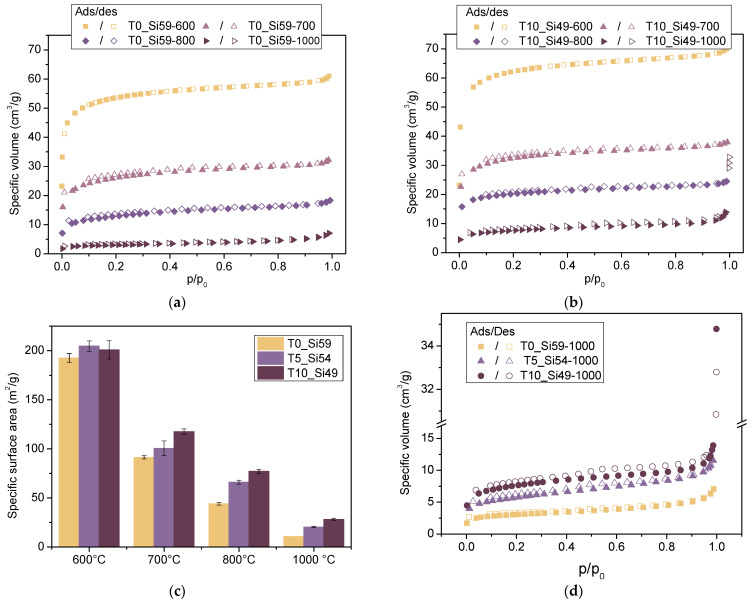
Nitrogen adsorption isotherms for samples (**a**) T0-Si59 and (**b**) T10-Si49. (**c**) BET-specific surface area for all of the samples at different pyrolysis temperatures (600–1000 °C). (**d**) Isotherms for the samples pyrolyzed at 1000 °C.

The effects of the composition and pyrolysis temperature on the microstructure of the pyrolyzed samples were analyzed using nitrogen adsorption–desorption isotherms and their respective BET specific surface areas (SSA) (Figure 5). The isotherms of the samples pyrolyzed between 600 and 800 °C presented pronounced adsorption volumes at very low relative pressures, corresponding to isotherm type I (b) [51], which is characteristic of materials with micropores and possibly narrow mesopores. A similar profile was observed for T10-Si49 pyrolyzed at 1000 °C. However, a moderate increase of the adsorption volumes and a slightly pronounced hysteresis was observed at relative pressures above 0.4 at a higher magnification (Figure 5d), which is a behavior closely related to isotherm Type IV [51], indicating a larger amount of mesopores. The sample T0-Si59_1000, on the other hand, presented the lowest adsorption volume, with increasing adsorption mainly in the range of the higher relative pressure region (isotherms type II), which is characteristic of macroporous materials (Figure 5d). The total amount of adsorbed gas correlates with the pore volume, and significantly decreases with the increase of the pyrolysis temperature.

Micro/mesoporosity was formed at intermediate pyrolysis temperatures (500–700 °C) due to the decomposition of the organic groups present in the preceramic polymers. High SSAs of up to 200 m^2^/g (T5_Si54-600, T10_Si49-600) were obtained for the tapes pyrolyzed at 600 °C, whereas at higher pyrolysis temperatures, due to the collapse of the micro/mesopores into larger pores, a gradual decrease in SSA was observed, reaching values as low as 10 m^2^/g (T0_Si49-1000).

The addition of filler particles can hinder the shrinkage of the bulk during the pyrolysis of preceramic polymers, providing paths for the release of the generated gases, thereby retarding the collapse of micro–mesopores [23]. In this work, the addition of increasing amounts of TiO_2_ nanoparticles into the membrane composition further preserved micro- and meso-porosity, leading to a higher specific surface area for all of the analyzed temperatures. Hojamberdiev et al. [52] also reported that the incorporation of TiO_2_/N-doped TiO_2_ into an SiOC matrix strengthened the porous structure against a gradual collapse at higher pyrolysis temperatures: as they increased the pyrolysis temperature from 700 to 900 °C, the SSA of the pure SiOC ceramic abruptly decreased from 398 to 60 m^2^/g, while less-pronounced reductions were observed for the SiOC/TiO_2_ (336 to 212 m^2^/g) and SiOC/N-doped TiO_2_ (254 to 129 m^2^/g) composites.

**Figure 6:**
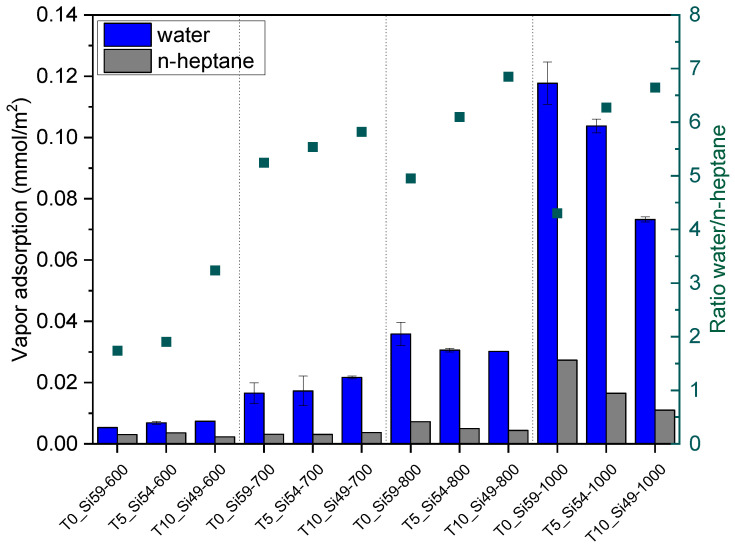
Water and n-heptane vapor adsorption, and the ratio of the maximum water and n-heptane adsorption (right axis) for all of the samples pyrolyzed at different temperatures (600–1000 °C).

In summary, the SSA of the PDC membranes can be tailored by adjusting the pyrolysis temperature and the particle size of the fillers used. Aside from the microporosity, the pyrolysis temperatures and the material composition can also have an influence on the surface properties of the membranes produced. Therefore, the hydrophilic/hydrophobic surface behavior of the produced tapes was evaluated by analyzing the adsorption of water and n-heptane vapor (Figure 6). In these measurements, a ratio of water-to-heptane >1 corresponds to a higher water uptake, indicating the higher hydrophilicity of the material, while a ratio < 1 indicates a higher affinity to n-heptane, and therefore a more hydrophobic character.

Figure 6 shows that the surface character is significantly influenced by the pyrolysis temperature. Although all of the samples presented a water-to-heptane ratio > 1, the samples pyrolyzed at 600 °C showed ratios 2–3-times lower than their counterparts pyrolyzed at higher temperatures. This can be explained by the decomposition of the polysiloxane during the pyrolysis process. As was already shown in the TGA analysis, the decomposition of the organic groups (phenyl and methyl) of preceramic polymers H44 and MK starts above 500 °C. In the samples pyrolyzed at 600 °C, the remaining functional groups provided a more hydrophobic character than in the samples pyrolyzed at higher temperatures, as the material loses its organosilicon character. Previous works [25,44,53] using the same preceramic polymers (with or without ceramer fillers) clearly demonstrated the hydrophobicity of materials pyrolyzed at temperatures of 600 °C by a water-to-heptane ratio of significantly <1. This indicates that not only the pyrolysis temperature but also the filler nature can play a role in the surface character of the produced samples. The use of pure SiC as a filler in a PDC matrix instead of ceramer fillers already showed an increase in the hydrophilicity of the samples. Moreover, the partial replacement of SiC by TiO_2_ nanoparticles (which are often applied to improve the hydrophilicity of polymeric membranes [54,55]) promoted a further increase in the water-to-heptane ratios. These results demonstrate that the microporosity and surface properties of polysiloxane-based material can be tuned according to the pyrolysis temperature and the nature of the filler particles in a single-step process. 

### 3.4. Adsorption and Photocatalytic Activity 

The XRD spectra described earlier confirmed that TiO_2_ was successfully incorporated into the membrane matrix in the anatase phase. However, the increase of the pyrolysis temperature from 600 to 1000 °C caused a reduction of the anatase peaks in the produced tapes (Figure 2). Thus, it is expected that the photocatalytic potential would gradually decrease in samples pyrolyzed at higher temperatures. 

**Figure 7:**
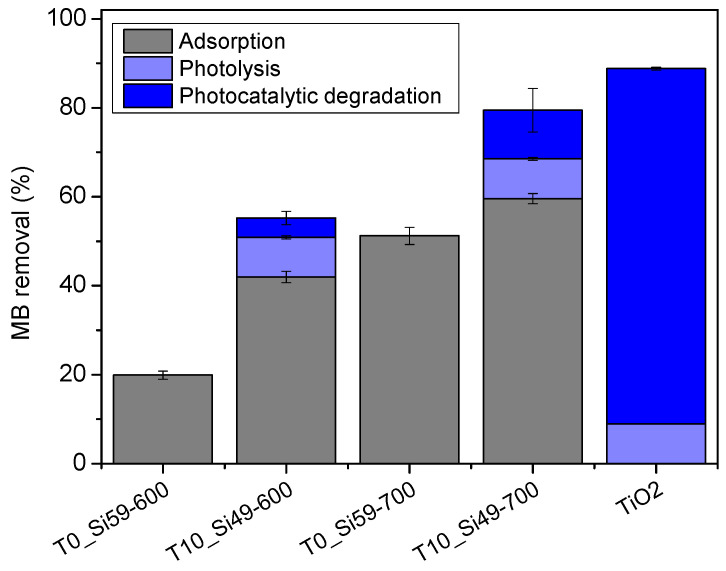
MB removal capacity of samples T0_Si59-600, T10_Si49-600, T0_Si59-700 and T10_Si49-700 after 24 h in the dark (adsorption), and under a UV light source (photocatalytic degradation) for the TiO_2_-incorporated samples (T10_Si49-600 and T10_Si49-700), for the equivalent amount of pure TiO_2_ nanoparticles, and in the absence of any material (photolysis).

The adsorption and the photocatalytic performances of the produced samples were assessed in a series of tests using methylene blue (MB) as a tracer dye and a UV light source. The initial results (Figure S3a) showed a significant adsorption of MB by the produced PDC material, and that the commonly used period of 2 h for adsorption in the dark [40,45,46] was not sufficient to reach equilibrium. Therefore, performing irradiation experiments after a short adsorption time in the dark would lead to misleading photocatalytic degradation capacities. In order to determine the maximum MB adsorption capacity of the prepared PDC material, and thus better separate the effects of adsorption and photocatalysis on the dye removal, samples T0_Si59-600, T10_Si49-600, T0_Si59-700 and T10_Si49-700 were exposed to MB solution (C_0_ = 50 mg/L) as a powder for 24 h. For comparison, the effects of the UV light source on MB were also determined in the absence of any material (photolysis), and in the presence of the equivalent amount of pure TiO_2_ nanoparticles. Figure 7 summarizes the outcome of this experiment.

In the evaluated period, the samples pyrolyzed at 700 °C presented a higher MB removal capacity than those pyrolyzed at 600 °C. As was previously demonstrated by the vapor adsorption and BET analysis of N_2_ isotherms, with increasing pyrolysis temperatures (ceramization process) the character of the surface of PDC materials becomes more hydrophilic, while at the same time SSA decreases. The higher MB adsorption of T10_Si49-700, despite its lower SSA than T10_Si49-600, could thus be related to its improved hydrophilic character, which allows the better wettability of the samples by the MB solution. The same trend can be observed for photocatalytic decomposition. The samples T10_Si49-600 and T10_Si49-700 removed 42% and 60% of MB, respectively, in the dark (adsorption), as opposed to 55% and 80%, respectively, under UV light. Taking into consideration that photolysis accounted for 9% of the reduction of the MB concentration, only 4% (T10_Si49-600) and 11% (T10_Si49-700) of the dye removal could be associated with photocatalytic degradation after a long period of UV light exposition. 

Previous studies in the literature [38,52,56] reported a more pronounced photocatalytic degradation of MB using TiO_2_ (particles or in situ formation) in PDC substrates, but the experimental designs of the study did not allow us to exclude with certainty the possibility that the observed MB removal was clearly separated from other influences, such as pure adsorption in the material. Bhattacharjee et al. [56] showed a superior rate of photocatalytic degradation of MB under UV light by anatase-loaded silica-based foams (developed using a mixture of H44, glass and Degussa P25 TiO_2_ nanoparticles, and a pyrolysis temperature of 800 °C) compared to plain TiO_2_ nanoparticles. However, the authors did not report a prior period of equilibrium-adsorption in the dark, or an analogous experiment without UV radiation. Hojamberdiev et al. [37] evaluated the adsorption and photocatalytic activity of TiO_2_/N-doped TiO_2_-incorporated SiOC composites in comparison with pure SiOC. In their study, the authors placed their samples in contact with an MB solution for 2 h in the dark in order to ensure the sufficient adsorption of dye molecules on the surfaces of the material prior to the UV irradiation. Their study, however, did not provide values or supporting data regarding the adsorption-equilibrium, and a higher reduction rate of MB was observed in the presence of the pure SiOC material than the one obtained solely by photolysis under UV irradiation, which indicates that the material might not have reached an adsorption-equilibrium in the reported period in the dark. A similar outcome could be expected for the TiO_2_/N-dopedTiO_2-_incorporated materials, especially when they present higher adsorption capacities than the pure SiOC, as suggested by the author.

In a different approach, Icin et al. [38] coated PDC nanobeads (pyrolyzed at 600 °C and 1200 °C) with titania precursor sol. The advantage of the coating procedure is that TiO_2_ is more readily accessible to light irradiation on the surface of the material than it is when entrapped in its matrix, while the disadvantage is the necessity of a secondary thermal process, which can represent an increase in the production cost. The authors [45] reported a high total MB removal efficiency of 97% (adsorption in the dark for 2 h, followed by photodegradation under UV for 4 h) for their TiO_2_-coated PDC material. Although the authors did not evaluate how much MB would be removed in a 6-h experiment without UV irradiation for comparison, they reported that the 2-h adsorption in the dark already accounted for 35% of the MB for the TiO_2_-coated samples. Therefore, similarly to our study, the efficiency of MB removal by photodegradation observed in different TiO_2_-incorporated/coated SiOC materials reported in the literature could also be due more to the adsorption phenomena in the PDC material than photocatalytic effects promoted by TiO_2_ under a proper light source.

The results of this study indicated that the possible anti-fouling effect on the produced PDC membranes from a photocatalytic reaction would be significantly low, as TiO_2_ particles are not sufficiently accessible when embedded in a PDC matrix, and are not easily quantified, as adsorption outshines the photodegradation effects. On the other hand, this significant adsorption capacity for MB, which continues for periods of time even longer than 24 h (Figure S3b), could be interesting for other wastewater treatment applications. The presence of TiO_2_ also seemed to influence the adsorption capacity of the samples. For the same pyrolysis temperature, the samples with 10% TiO_2_ presented a higher removal of MB than their TiO_2_-free counterparts. This outcome could be attributed to their higher SSA and improved hydrophilic character; the latter was observed especially for the samples T0_Si59-600 and T10_Si49-600. Despite the similar SSA, respectively 192.5 ± 4.5 and 200.8 ± 9.3 m^2^/g, the more hydrophilic TiO_2_-incorporated material removed 20% more MB in the evaluated period of 24 h. The MB adsorption capacity of the PDC material evaluated in this study was comparable to other adsorbents described in the literature (Table 1), and its potential should be further investigated, e.g., for dye-removal applications. 

### 3.5. Mechanical Strength

**Figure 8:**
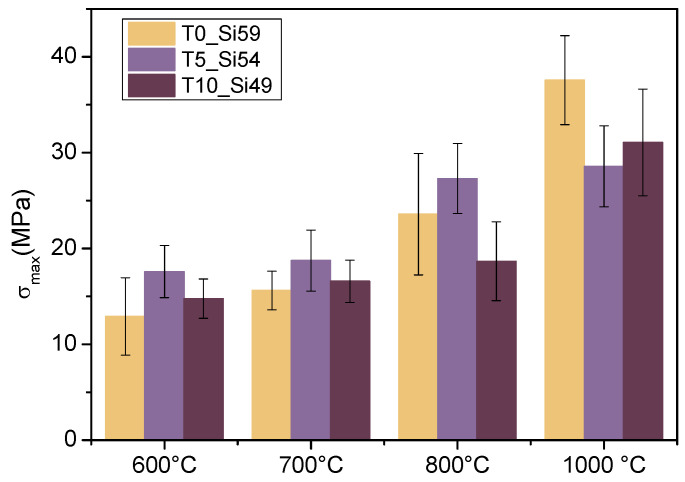
Flexural strength (σ_max_) for tapes T0_Si59, T5_Si54, and T10_Si49 pyrolyzed at 600–1000 °C.

The mechanical resistance of the membranes was accessed via a three-point bending test in order to evaluate the maximum flexural strength (σ_max_) of the pyrolyzed tapes (Figure 8). An enhanced mechanical resistance with an increase of the pyrolysis temperature is expected as the preceramic precursor undergoes the ceramization process [2]. 

Hybrid ceramics are formed in the temperature interval of 600–800 °C, and are characterized by the gradual disappearance of the organosilicon polymer nature of the material due to thermal degradation in an inert atmosphere, while the precursor completely transforms into an amorphous ceramic material at 1000 °C. Therefore, a gradual increment was observed for the flexural strength. Aside from this, as previously explained, the incorporation of filler particles in PDC materials controls the shrinkage and prevents the formation of macro-defects derived from the gas release during the pyrolytic polymer-to-ceramic conversion. In this study, the amounts of SiC and TiO_2_ particles were varied; however, the total amount of solid loading was kept constant. The average σ_max_ for the different membrane compositions did not change significantly at the same pyrolysis temperature. The partial replacement of SiC by TiO_2_ particles neither improved nor affected the mechanical resistance of the pyrolyzed tapes. An overall increment of σ_max_ 15 ± 5 MPa to 35 ± 5 MPa was observed as the pyrolysis temperature was increased from 600 to 1000 °C. 

Various ceramic membranes described in the literature (Table 2) with flexural strength values between 15–40 MPa have already been considered suitable for the microfiltration process (with a usual transmembrane pressure interval of 0.1–2 bar). Thus, the ceramic hybrid membranes produced in this work, including those pyrolyzed at lower temperatures (600–700 °C), exhibited comparable flexural strength to the ceramic microfiltration membranes described in the literature (Table 1), and to the SiOC membranes produced by Dong et al. [41], in particular. The authors also evaluated the effect of pyrolysis on their SiOC membranes produced in a higher temperature range of 1100–1400 °C. They observed an increase in flexural strength from 19 ± 1 to 23 ± 2 MPa when the temperature increased from 1100 to 1200 °C; however, a further increase in temperature to 1400 °C significantly reduced the mechanical resistance of the membrane (4 ± 1 MPa). According to the authors, the mixed bonding between O, C, and Si atoms in the amorphous structure at 1200 °C played an important role in the thermodynamic stability of the ceramic structure. At 1400 °C, the accelerated phase separation resulted in a decrease of mixed bonds and an increase of interfacial regions, which caused more free carbon fragments, which led to the decay of the samples pyrolyzed at high temperatures. A more detailed analysis of the structural evolution of the silica domains, SiC domains, and free carbon regions, as well as the dominant factors of the stabilization and destabilization of the pyrolyzed polysiloxane-based structures, can be found elsewhere [62]. Overall, these studies suggest that the directly proportional relationship between increasing pyrolysis temperatures and enhanced mechanical resistances in pyrolyzed polysiloxane-based materials is true up to a certain point, from which further temperatures increments could lead to lower mechanical resistance due to the structural evolution during the transition from an amorphous to a crystalline structure. Therefore, the pyrolysis temperature is an important parameter that needs to be optimized. 

In this work, the lower processing temperatures used can represent an economic advantage in the production cost of microfiltration membranes; thus, the potential of the produced membranes for filtration applications is worth further exploration. 

### 3.6. Membrane Performance

**Figure 9:**
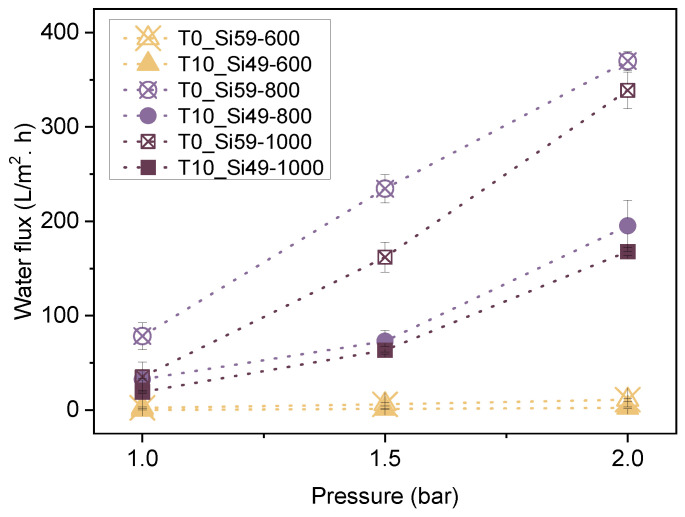
Water flux as a function of the applied pressure (bar) for selected membranes in a dead-end configuration.

The water permeation and oil-in-water (O/W) emulsion filtration performance of the membranes was assessed using a homemade setup in a dead-end configuration. Figure 9 shows the water permeation performance of the membranes T0_Si59 and T10_Si49, which were pyrolyzed at different temperatures of 600, 800 and 1000 °C, and at different pressures (1, 1.5 and 2 bars).

An expected linear relationship between the water flux and the applied pressure was observed for all of the samples with higher pressures, resulting in higher permeation fluxes. The pore size and surface character of the samples also played a role in the permeation performance. Bigger pore sizes represent lower mass transfer resistances, resulting in higher permeation rates. In this study, the highest fluxes of pure water were obtained for the samples T0_Si59-800 (370 L/m^2^·h) and T0_Si59-1000 (340 L/m^2^·h) at 2 bar, while the lowest fluxes were observed for samples T0_Si59_600 (11 L/m^2^·h) and T10_Si49_600 (2.5 L/m^2^·h). Aside from the smaller average pore size, the lower permeation observed for samples pyrolyzed at 600 °C can also be attributed to the more hydrophobic character of these membranes, which has already been described in Section 3.3. A comparison between two samples with similar average pore sizes but different surface characteristics, T0_Si59_600 (Ø = 0.6 µm) and T10_Si49_1000 (Ø = 0.7 µm), illustrated the superior water permeation performance of hydrophilic membranes at 2 bar, at which the water flux of T10_Si49_1000 is over 15-times higher than that of T0_Si59_600.

The performances of the membranes T0_Si59-800, T10_Si49-8000, T0_Si59-1000, T10_Si49-1000 in the separation of an oil-in-water emulsion (C_0_ = 1000 mg/L) was determined at a fixed pressure of 2 bar. The samples T0_Si59-600 and T10_Si49-600 could not be evaluated due to their hydrophobic character and the associated high water permeation resistance. Figure S4 shows the size distribution of the oil droplets in the prepared emulsion, which was analyzed by laser diffraction. The oil droplet diameter ranged between 1 and 20 µm, with a volume median diameter (D_(v,0.5)_) of 5.3 µm (D_(v,0.1)_ = 2.9 µm, D_(v,0.9)_ = 10.5 µm). Oily wastewaters containing oil droplets with a size smaller than 20 µm—referred to as emulsified oil—are usually stable, and can therefore be treated more effectively with membrane technology than with other conventional separation processes, such as centrifugation and coagulation [2]. In this study, the average pore diameters of the produced membranes T0_Si59-800, T10_Si49-8000, T0_Si59-1000 and T10_Si49-1000 were smaller than those of most oil droplets, indicating that the selected membranes are good candidates for the purification of the prepared emulsion. 

**Figure 10:**
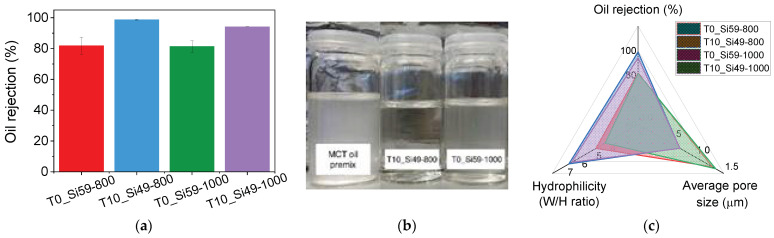
(**a**) MCT oil rejection for samples T0_Si59-800, T10_Si49-800, T0_Si59-1000 and T10_Si49-1000. (**b**) MCT oil emulsion before and after filtration with membranes T10_Si49-8000 and T0_Si59-1000. (**c**) Radar plot with the different parameters influencing the oil rejection.

High average oil rejection coefficients were obtained for the membranes T10-Si49_800 (98.8%) and T10-Si49_1000 (94.1%), while separation efficiencies of 81.8% and 81.4% were observed for T0-Si59_800 and T0-Si59_1000 (Figure 10). The higher oil rejection ratios of T10_Si49-8000 and T10_Si49-1000 can be attributed to their narrower pore size distribution (Ø = 0.7 µm), indicating that the pore diameters of these membrane are small enough to efficiently prevent most oil droplets from permeating. Additionally, with respect to the surface characteristics of the membranes, it has already been shown that the TiO_2_-containing samples are more hydrophilic than their TiO_2_-free counterparts pyrolyzed at the same pyrolysis temperature, which contributes to their better separation performance. Figure 10c shows an overview of the relationships. 

More generally, the oil separation performance of all of the membranes evaluated is comparable to that of various ceramic membranes reported in the literature (Table 1). The lower processing temperatures of PDC membranes can provide an economic advantage in the production costs of microfiltration membranes; therefore, their use in this application should be further explored.

## 4. Conclusions

Symmetric porous ceramer and ceramic microfiltration membranes with narrow pore size distributions were produced via the tape-casting technique, using polysiloxanes as preceramic precursors. We demonstrated the ways in which the membranes’ properties can be tailored according to the pyrolysis temperature and the incorporation of different filler particles. The incorporation of TiO_2_ in the SiC/SiOC composites further preserved the transient micro-mesoporosity generated during pyrolysis, resulting in a higher SSA. Additionally, the lower pyrolysis temperature allowed the incorporation of TiO_2_ in its photocatalytically active phase. However, it could be shown that the adsorption phenomena in the PDC material outshone the photocatalytic effects promoted by TiO_2_ using a proper light source, and that the improper assessment of adsorption without irradiation could result in misleading photodegradation capacities. Nevertheless, the significant adsorption capacity for MB of the produced PDC material, which further increased with the incorporation of TiO_2_ material, shows potential for other applications in wastewater treatment, and should be further investigated. With regard to microfiltration applications, the ceramer and ceramic membranes produced showed adequate flexural strength which was comparable to that of various ceramic membranes for microfiltration described in the literature, which were sintered at higher temperatures. For the new membranes, high oil removal efficiencies of 81–98% were obtained for an initial oil concentration of 1000 mg/L, and were controlled predominantly by adjusting the mean pore diameter and membrane hydrophilicity. Additionally, the lower processing temperatures could represent an economic advantage in the production cost of microfiltration membranes.

## Figures and Tables

**Table 1:** Adsorption capacity of MB on different adsorbents.

Material	Adsorption Capacity (mg/g)	Ref.
Fly ash	13.4	[57]
Granular active carbon	21.5	[58]
Neem leaf	8.8/19.6	[59]
Rice biomass8	8.1	[60]
SNCM *	20.0	[61]
T10_Si49-700	9.3 (24 h) ** 15.2 (96 h) ***	This study
T10_Si49-600	6.6 (24 h) ** 12.8 (96 h) ***	This study

* Synthetic layered sodium silicate magadiite nanosheets. ** Langmuir adsorption capacity (24 h). *** 96 h Kinetic experiment, C_0_ = 50 mg/L.

**Table 2:** Properties of planar ceramic membranes for microfiltration.

Membrane Material	Sintering Temp. (°C)	Porosity (%)	Pore Size (µm)	Thickness (mm)	Pressure (bar)	Flexural Strength (MPa)	Oil Conc. (mg/L)	Oil Rej. (%)	Ref.
Al_2_O_3_	1350	47	0.4	5	1	15	284	99.9	[63]
Al_2_O_3_	1300	37	0.5	3	2	22	200	99	[64]
Al_2_O_3_ + fly ash-mullite	1050	34.5	0.1	3	0.5	30	-	-	[65]
Moroccan clay	950	31–40	1.5–2.8	1.5	0–0.12	14–16	-	-	[66]
Moroccan clay/phosphate.	1100	28	2.5	1.6	0.12	17.5	-	-	[67]
Mullite whisker (MoO_3_)	1400	47	0.19	1.5	0.5–2	34 ± 4	250	97	[68]
Monolithic mullite	1400	64	0.3	-	2	42 ± 5	200–1000	96	[69]
Silicate/clay-mineral	1000 1050 1100	32 33 34	0.29 0.37 0.67	3	3	32 ± 3 30 ± 5.5 28 ± 5	600	86	[70]
SiOC	1200	42	0.59	0.65	0.5–2.0	23 ± 2	1000	94.6	[41]
TiO_2_/clay/quartz/feldspar	950	37–52	0.8–1.0	2	0.7–3.5	28–33	50–200	70–99	[54]
Si_3_N_4_	1650	46–56	0.61	-	1–2	51–105	1000	83–88	[71]

## Data Availability

The data that support the findings of this study are available on request from the corresponding author.

## Supplementary Materials

The following supporting information can be downloaded at www.mdpi.com/article/10.3390/membranes12020175/s1. Table S1: Membrane composition; Figure S1: Thermal Gravimetric analysis for samples T0_Si59, T5_Si54 and T10_Si49; Figure S2.: SEM images of the cross-section areas of samples T0_Si59-700 (**a**,**b**) and T10_Si59-700 8 (**c**,**d**) revealing symmetrical sponge-like structures; Figure S3: (**a**) MB adsorption isotherms of the sample T10-700 as a powder and as a tape, and (**b**) MB removal over time using samples T10-600 and T10-700 as powders and as tapes (96 h); Figure S4: Droplet size distribution of the feed O/W emulsion (MCT oil, C_0_ = 1000 mg/L).
