# Supplementary material for: Influence of the Pyrolysis Temperature and TiO2-Incorporation on the Properties of SiOC/SiC Composites for Efficient Wastewater Treatment Applications
Source: Membranes (Basel). 2022 Feb 2;12(2):175. doi: 10.3390/membranes12020175 (PMC8875749; doi:10.3390/membranes12020175)
Supplement: Supplementary file 1 [file membranes-12-00175-s001.zip › membranes-1565836-supplementary.pdf]

membranes

Article

Supplementary Materials: Influence of Pyrolysis Temperature and TiO_2-Incorporation on Properties of SiOC/SiC Composites for Efficient Wastewater Treatment Applications

Natália C. Fontão [1], Lucas N. Ferrari [1,2], Joice C. Sapatieri [1,3], Kurosch Rezwan [1,4] and Michaela Wilhelm [1,*]

[1] Advanced Ceramics, University of Bremen, 28359 Bremen, Germany
[2] Department of Mechanical Engineering, Federal University of Santa Catarina, Florianopolis 88040-900, Brazil
[3] Department of Chemical Engineering and Food Engineering, Federal University of Santa Catarina, Florianopolis 88040-900, Brazil
[4] MAPEX—Centre for Materials and Processes, University of Bremen, 28359 Bremen, Germany
* Correspondence: mwilhelm@uni-bremen.de

Citation: Fontão, N.C.; Ferrari, L.N.; Sapatieri, J.C.; Rezwan, K.; Wilhelm, M. Influence of Pyrolysis Temperature and TiO_2-Incorporation on Properties of SiOC/SiC Composites for Efficient Wastewater Treatment Applications. *Membranes* **2022**, *12*, 175. https://doi.org/10.3390/membranes12020175

Academic Editor: Alfredo Cassano

Received: 5 January 2022
Accepted: 28 January 2022
Published: 2 February 2022

Publisher's Note: MDPI stays neutral with regard to jurisdictional claims in published maps and institutional affiliations.

Copyright: © 2022 by the authors. Licensee MDPI, Basel, Switzerland. This article is an open access article distributed under the terms and conditions of the Creative Commons Attribution (CC BY) license (https://creativecommons.org/licenses/by/4.0/).

Supplementary Data

Table S1. Membrane composition

Sample	MK (g)	H44 (g)	AZO (g)	SiC (g)	TiO_2 (g)	Imidazole (g)
T0_Si59	2.0	2.0	3.0	5.9	0	0.1
T5_Si54	2.0	2.0	3.0	5.4	0.5	0.1
T10_Si49	2.0	2.0	3.0	4.9	1.0	0.1

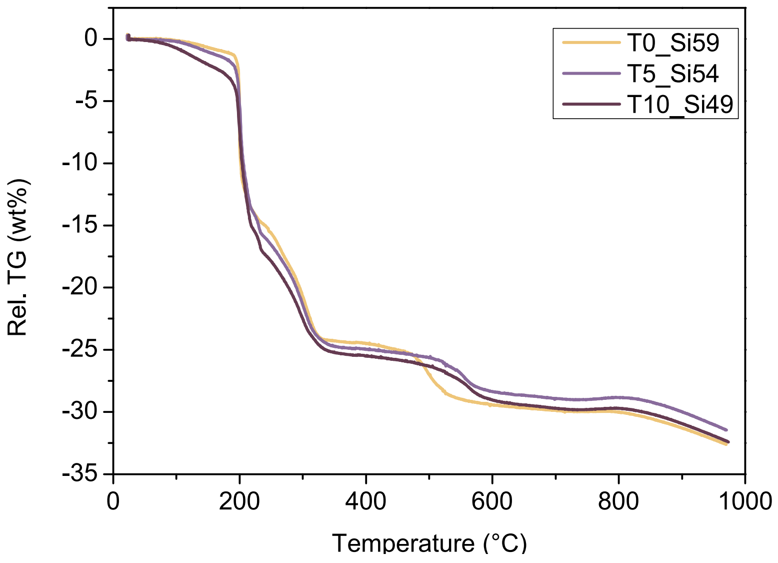

Figure S1. Thermal Gravimetric analysis for samples T0_Si59, T5_Si54 and T10_Si49.

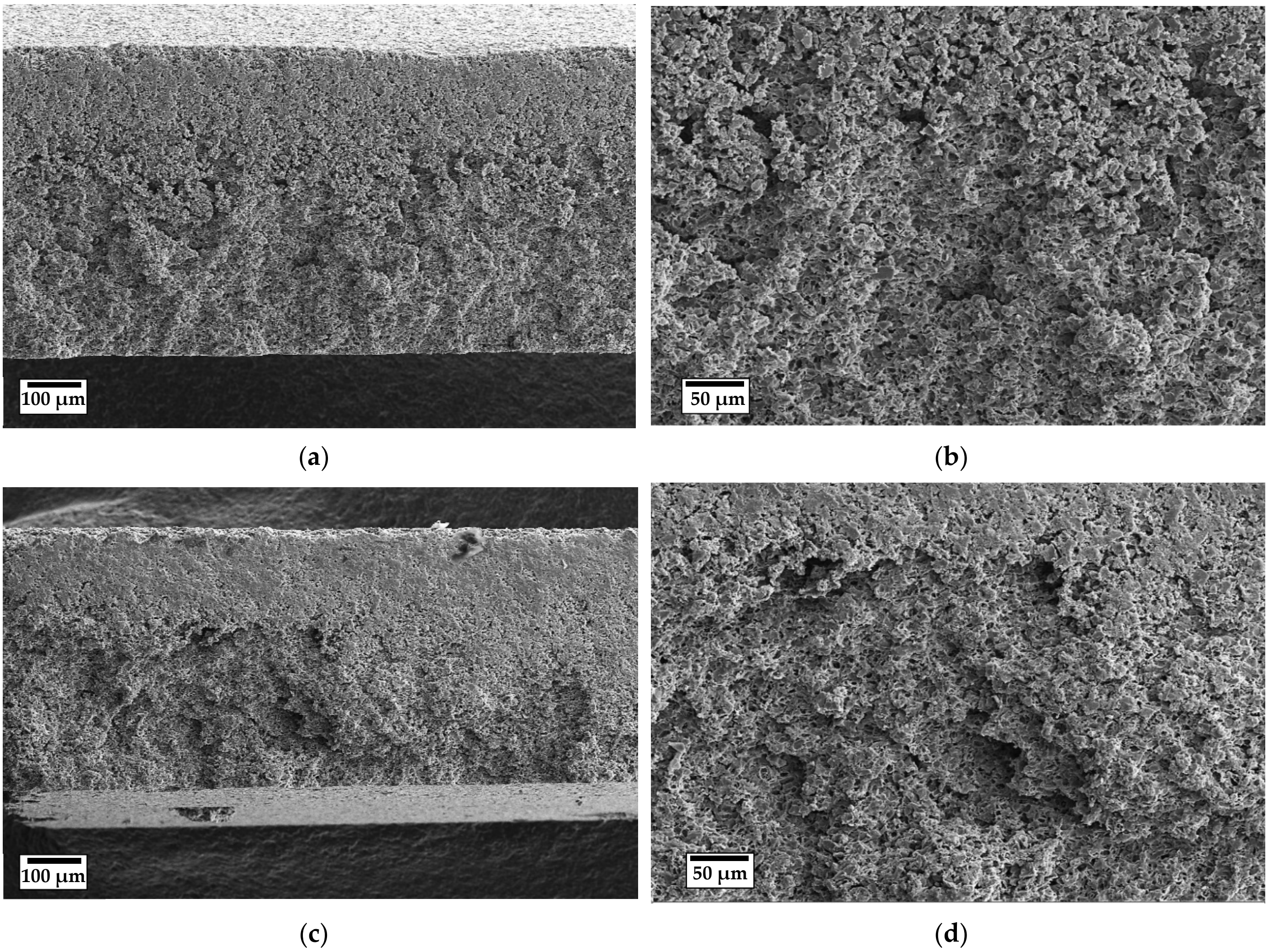

Figure S2. SEM images of the cross-section areas of samples T0_Si59-700 (**a**,**b**) and T10_Si59-700 8 (**c**,**d**) revealing symmetrical sponge-like structures.

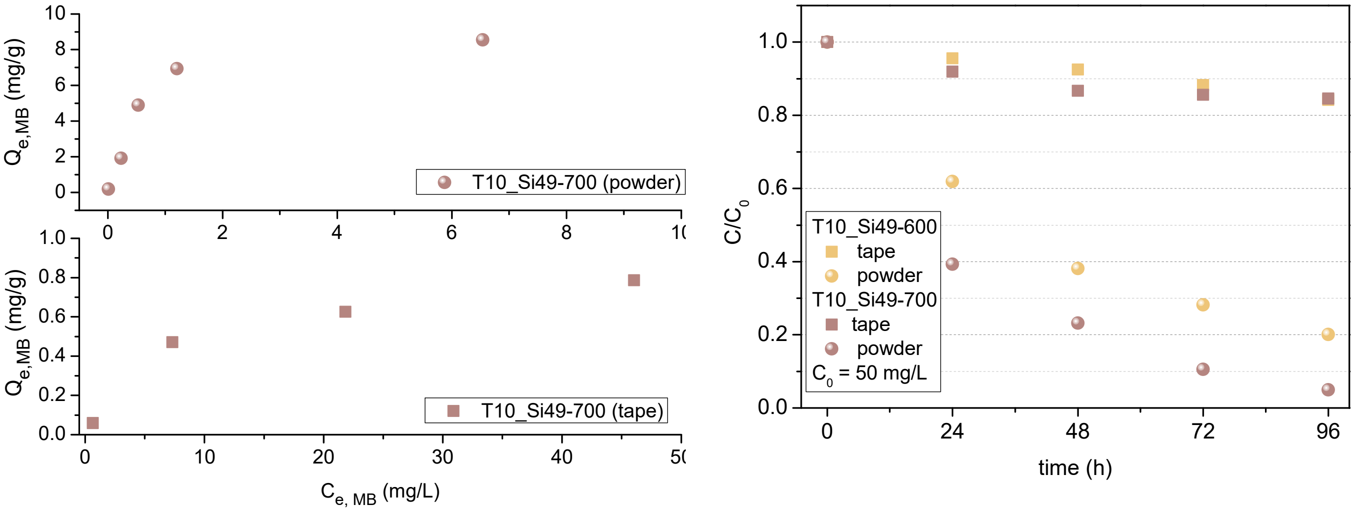

(**a**) (**b**)

Figure S3. (**a**) MB adsorption isotherms of the sample T10-700 as a powder and as a tape. (**b**) MB removal over time using samples T10-600 and T10-700 as powders and as tapes (96 h).

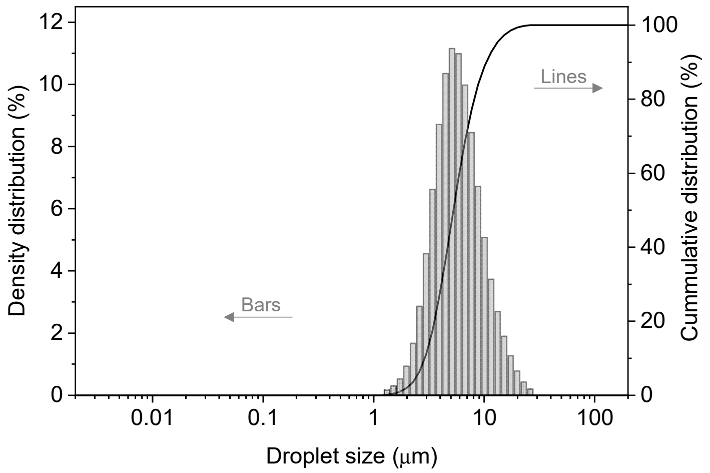

Figure S4. Droplet size distribution of the feed O/W emulsion (MCT oil, C0 = 1000 mg/L).
